# Inhibitory Effects of Macelignan on Tau Phosphorylation and Aβ Aggregation in the Cell Model of Alzheimer's Disease

**DOI:** 10.3389/fnut.2022.892558

**Published:** 2022-05-18

**Authors:** Liang Gu, Nan Cai, Meiting Li, Decheng Bi, Lijun Yao, Weishan Fang, Yan Wu, Zhangli Hu, Qiong Liu, Zhijian Lin, Jun Lu, Xu Xu

**Affiliations:** ^1^Shenzhen Key Laboratory of Marine Bioresources and Ecology and Guangdong Provincial Key Laboratory for Plant Epigenetics, College of Life Sciences and Oceanography, Shenzhen University, Shenzhen, China; ^2^Digestive Diseases Center, The Seventh Affiliated Hospital, Sun Yat-sen University, Shenzhen, China; ^3^School of Science and School of Interprofessional Health Studies, Faculty of Health and Environmental Sciences, Auckland University of Technology, Auckland, New Zealand; ^4^Instrumental Analysis Center, Shenzhen University, Shenzhen, China; ^5^Department of Neurology, Peking University Shenzhen Hospital, Shenzhen, China; ^6^School of Public Health and Interdisciplinary Studies, Faculty of Health and Environmental Sciences, Auckland University of Technology, Auckland, New Zealand; ^7^Maurice Wilkins Centre for Molecular Discovery, Auckland, New Zealand

**Keywords:** Alzheimer's disease, macelignan, AMPK pathway, autophagy, PERK/eIF2α pathway

## Abstract

Alzheimer's disease (AD) is a neurodegenerative disorder mainly affecting old population. In this study, two Tau overexpressing cell lines (SH-SY5Y/Tau and HEK293/Tau), N2a/SweAPP cell line, and 3× Transgene (APPswe/PS1M146V/TauP301L) mouse primary nerve cell lines were used as AD models to study the activity and molecular mechanism of macelignan, a natural compound extracted from *Myristica fragrans*, against AD. Our study showed that macelignan could reduce the phosphorylation of Tau at Thr 231 site, Ser 396 site, and Ser 404 site in two overexpressing Tau cell lines. It also could decrease the phosphorylation of Tau at Ser 404 site in mouse primary neural cells. Further investigation of its mechanism found that macelignan could reduce the phosphorylation of Tau by increasing the level of autophagy and enhancing PP2A activity in Tau overexpressing cells. Additionally, macelignan could activate the PERK/eIF2α signaling pathway to reduce BACE1 translation, which further inhibits the cleavage of APP and ultimately suppresses Aβ deposition in N2a/SweAPP cells. Taken together, our results indicate that macelignan has the potential to be developed as a treatment for AD.

## Introduction

Alzheimer's disease (AD) is a neurodegenerative disorder with a global public fitness priority identified by the World Health Organization (1). In the past few decades, four United States Food and Drug Administration (FDA)-approved medications have been used for managing cognitive impairment and dysfunction symptoms of AD (2). However, these clinical medicines can only alleviate the symptom of AD rather than remedy it (3). There are presently >100 compounds in AD clinical trials (4). Although a lot have been known about the pathogenesis since the first report of AD in 1907 (5), there is no effective treatment to date. Searching for new treatment is still an on-going task for scientists and physicians.

The pathological features of AD encompass amyloid plaques, neurofibrillary tangles (NFTs), and neuroinflammation (6). Amyloid plaques and NFTs are typical pathological facets. NFTs mainly consist of intracellular paired helical filaments (PHFs) of the abnormal hyperphosphorylated form of tau protein (7). Under normal circumstances, Tau promotes the assembly and steadiness of microtubules and the transport of vesicles in neuro cells (8). When Tau encounters the released kinase, it is hyperphosphorylated. The hyperphosphorylated Tau further leads to Tau oligomerization. These hyperphosphorylated Tau are converted to a range of filaments, such as PHFs, which aggregate into NFTs (2). The existence of NFTs in the cytoplasm of neurons leads to the loss of communication and signal processing between neurons, causing neuronal apoptosis (9). Based on this, methylene blue has been used in an AD clinical trial with promising outcomes (10). The mechanism study in a tauopathy mouse model suggests that methylene blue could reduce Tau fibrillization and aggregation, and induce autophagy to alleviate neuron dying (11).

Amyloid plaques are formed through the precipitation of amyloid β-protein (Aβ) outside nerve cells. Aβ peptide is produced when β-amyloid precursor protein (APP) is sequentially cleaved by β-secretase and γ-secretase. The aberrancy cleavage of APP and the mutations of γ- and β-secretase all account for the abnormal manufacturing of Aβ peptide, which may increase the neuron loss and synaptic damage, and amyloid plaques (12). Many pharmaceutical companies have committed resources to develop Aβ-targeted drugs. However, many compounds, such as Verubecestat and Avagacestat, have failed in phase 3 clinical trials. Owing to the elusive pathogenesis of AD, the single-target anti-AD drugs appear to be unsuccessful based on failed clinical trials (13, 14). Hence, multi-target drugs are now proposed to fight against AD (15).

Natural compounds are gaining attention due to their bioactivities. *Myristica fragrans* (*M. fragrans*) is a tropical evergreen tree native to Indonesia and cultivated in India, Iran, the West Indies, and South America. Mace is the seed of nutmeg plant, *M. fragrans*, containing components such as flavonoids, neolignans, and lignans. Among these components, lignan has shown great therapeutic potential in various diseases (16). Macelignan (MLN, CAS 107534-93-0) is a sort of lignan derived from *M. fragrans* mace. Pharmacological studies have shown that MLN possesses a broad range of properties, such as anti-inflammatory (17), anti-cancer (18), anti-bacterial (19), and hepatoprotective activity (20). Studies have found that MLN could effectively reduce the hippocampal microglial activation induced by lipopolysaccharide in rat's brain, and suppress the spatial memory impairments caused by lipopolysaccharide in mice (21). Nevertheless, MLN's effect on the pathophysiology of AD is still unknown.

In this study, the anti-AD activity of MLN and its possible mechanism of action were investigated through Tau hyperphosphorylation and Aβ production in a number of AD cell models.

## Materials and Methods

### Materials

MLN was bought from Selleck Chem (Houston, TX, United States). Thioflavin T (ThT), Dimethyl Sulfoxide, Paraformaldehyde (PFA) and 4',6-Diamidino-2-Phenylindole (DAPI) were purchased from Sigma-Aldrich (St. Louis, MO, USA). Fetal bovine serum (FBS) was procured from Gibco (Grand Island, NY, USA). Gluta MAX, L-glutamine, B27 supplement, Dulbecco's Modified Eagle's medium (DMEM), trypsin, opti-MEM, penicillin, neurobasal medium, MEM Non-Essential Amino Acids Solution (MEM NEAA), and streptomycin were purchased from Biological Industries (Kibbutz Beit Haemek, Israel). Bicinchoninic acid (BCA), cell counting kit (CCK)-8 and cell lysis buffer were obtained from KeyGen Biotech (Nanjing, Jiangsu Province, China). Phosphate-buffered saline (PBS) and Hank's balanced salt solution (HBSS) were purchased from Hyclone (Logan, UT, USA). Antibodies of AMP-activated protein kinase [AMPK (2532)], p-AMPK (2535), liver kinase B1 [LKB1 (3047)], p-LKB1 (3482), silent information regulator of transcription 1 [SIRT1 (9475)], p62 (5114), Beclin-1 (3495), mechanistic target of rapamycin [mTOR 2983)], p-mTOR (2971), p70 S6K (2708), p-p70 S6K (9205), microtubule-associated protein II light chain3 [(LC3, 12741)], eIF2α (9722), p-eIF2α (9721), PERK(3192), p-PERK(3179) and β-actin (3700) were procured from Cell Signaling Technology (Beverly, MA, USA). Antibodies of Tau-5 (ab80579), pS396-Tau (ab109390), pS404-Tau (ab92676), pT231-Tau (ab151559), MAP2 (ab5392), PP2Aα (ab137825), PP2Aα+β (ab32104), APP (ab32136), BACE1 (ab2077) and horseradish peroxidase (HRP)-conjugated secondary antibodies were bought from Abcam (Cambridge, UK). Antibodies of Aβ (803002) was procured from Biologend (Signet, USA). Bovine serum albumin (BSA) and other chemical reagents used in this study were purchased from Shanghai Macklin Biochemical (Shanghai, China).

### Cell Culture

SH-SY5Y/Tau cells were durably transfected with the longest human Tau 441 cDNA from human neuroblastoma SH-SY5Y cells (Shanghai Cell Bank of the Chinese Academy of Sciences, Shanghai, China). DMEM/F-12 medium blended with 10% FBS, 1% GlutaMAX, 1% MEM NEAA and 1mM sodium pyruvate were used to culture SH-SY5Y/Tau cells. Human embryonic kidney 293 (HEK293) cells with stable expression of the longest human Tau 441 cDNA were created and named as HEK293/Tau, a gift from Professor Jianzhi Wang of Tongji Medical College, Huazhong University of Science and Technology. HEK293/Tau cells were cultured in the medium containing 90% DMEM, 10% FBS, and G-418 antibiotics (0.2 mg/mL). Swedish mutant APP overexpressed in mouse neuroblastoma N2a cells. The cells have been named N2a/SweAPP cells. The cell line was a gift and provided by Professor Yunwu Zhang from Xiamen University. N2a/SweAPP cells were cultured in 50% DMEM medium blended with 40% opti-MEM medium and 10% FBS. All cell culture media were added with 100 unit/mL penicillin and 100 μg/mL streptomycin. All cells were maintained under a humidified environment of 95% air and 5% CO_2_ at 37 °C.

### Primary Neuron Acquisition and Culture

Mice were stably transfected with mutant human APPswe,TauP301L genes and mutant mice gene PS1M146V (called 3 × Transgene-AD mouse) were bought from the Jackson Laboratory (JAX order number 3591206, Bar Harbor, ME, USA). The 3 × Transgene-AD mice were bred at 12 h mild 12 h darkish stipulations of specific pathogen-free (SPF) circumstances. All animal experiments were conducted with the approval of the Laboratory Animal Ethics Committee of Shenzhen University (Permit Number:SYXK 2014-0140) and in accordance with the guidelines and regulations for animal experiments. The hippocampus of the new-born (within 24 h) 3 × Transgene-AD mice was used to obtain the primary neuron cells. The pre-cold HBSS was used to wash the dissected hippocampal tissues, then hippocampal tissues were cut into small portions using a surgical blade. These fragments were transferred to a new plate containing papain (2 mg/mL) and digested for 30 min at 37°C. The digested cells were filtrated and then centrifuged at 1,000 *g* for 5 min. After discarding the supernatant, the cell pellets were resuspended in medium and plated on poly-L-lysine (0.1 mg/mL)-coated plates. A neurobasal medium containing 2% B27 supplement, 1% L-glutamine, 100 unit/mL penicillin, and 100 μg/mL streptomycin was used to culture the primary neuron cells in a humidified 5% CO_2_ environment at 37°C.

### Cell Viability Assay

Cells (5 × 10^3^ cells/well) were incubated in a 96-well plate with different concentrations (0, 10, 15, and 20 μM) of MLN for 24 h. After the treatment, supernatant was removed, and 100 μL solution containing 10% CCK-8 solution and 90% DMEM medium was added to each well. The absorbance of each well was measured at 450 nm using a Spectra Max microplate reader (Thermo Scientific, Hudson, NH, USA) after 2 h incubation at 37°C.

### Immunofluorescence Assay

Cells were uniformly seeded in confocal dishes, incubated overnight, and then stimulated with 20 μM MLN for 24 h. Cold PBS was used to rinse the cells for three times and then 4% PFA was added to cells at room temperature for 15 min. Cells were blocked by PBS solution containing 10% (w/v) goat serum and 0.1% Triton X-100 at room temperature for 1 h. After that, primary antibodies were added to the cells and incubated for 24 h at 4°C. Cells were then washed thrice with PBS and incubated for 1 h with Alexa Fluor-conjugated secondary antibody at the room temperature. Cell nuclei were stained with DAPI. Confocal microscope (Carl Zeiss, Thornwood, NY, USA) was used to examine the stained samples. Images were acquired and processed by using the ImageJ software (https://imagej.nih.gov/ij/).

### Transmission Electron Microscopy Analysis

Samples containing R3-Tau (20 μM), and heparin (16 μM) were incubated for 24 h at 37 °C with or without MLN (20 μM). After the incubation, each sample was blanketed on a 230-mesh copper grid (Beijing Zhongjingkeyi Technology Co.) and incubated at room temperature for 2 min. Then, after washed twice with distilled water, 5 μL of 1% uranyl acetate (w/v, H_2_O) was dropped on the copper mesh and stained for 1 min. Filter paper was used to remove unbond uranyl acetate. Samples were dried at room temperature. A JEM-1230 transmission electron microscope (JEOL, Tokyo, Japan) was used to obtain images of the samples at a magnification of 50,000x.

### Thioflavin-T Fluorescence Assay

Freshly prepared R3-Tau, heparin, and ThT were dissolved in 50 mM PBS and blended with 5, 10, 15, 20 μM MLN, or Dimethyl Sulfoxide (Ctrl group). And Blank group was added with heparin, Dimethyl Sulfoxide and ThT. Each well of the cell seeding 96-well plate was added with 200 μL of the above mixture and incubated at 37°C. A microplate reader (Fluoroskan Ascent FL, Thermo Scientific) was used to excite the samples at 440 nm and record absorbance at 485 nm filter. The intensity of the fluorescence of each sample at one-of-a-kind time points was recorded.

### Western Blot Analysis

After being treated with MLN, cells were gathered and washed with cold PBS three times. Cell lysis buffer containing 1% PMSF (100 mM), 1% protease, and phosphatase inhibitor was added to the cells on ice for 30 min. Protein concentrations of lysates were measured using the BCA kit. Proteins were then separated through SDS-PAGE. After strolling of SDS-PAGE, the proteins were transferred onto a nitrocellulose filter (NC) membrane (Merch/Millipore, Schwalbach, Germany). The NC membranes were blocked with 5% BSA solution for 1 h and incubated with primary antibodies for 24 h at 4°C. TBST (Tris-buffered saline, 0.1% Tween 20) solution was used to wash the membranes for three times. HRP-conjugated secondary antibody was added and incubated at the room temperature for 1 h. After washing membranes three times with TBST, the specific binding proteins were shown by LAS3000 luminescent image analyzer (Fujifilm Life Science, Tokyo, Japan) after adding an enhanced chemiluminescence solution.

### Statistics

Results were presented as mean ± standard deviation (SD) of at least triplicate independent experiments in every group. Statistical analyses were carried out by using the GraphPad Prism 5.0 software (GraphPad Software, San Diego, CA, USA). Unpaired Student's-test was used for the comparison of two groups. The values of ^*^ *p* < 0.05, ^**^ *p* < 0.01, and ^***^ *p* < 0.001, stand for statistically significant differences.

## Results

### MLN Suppressed the Aggregation of Tau *in vitro*

**Figure 1:**
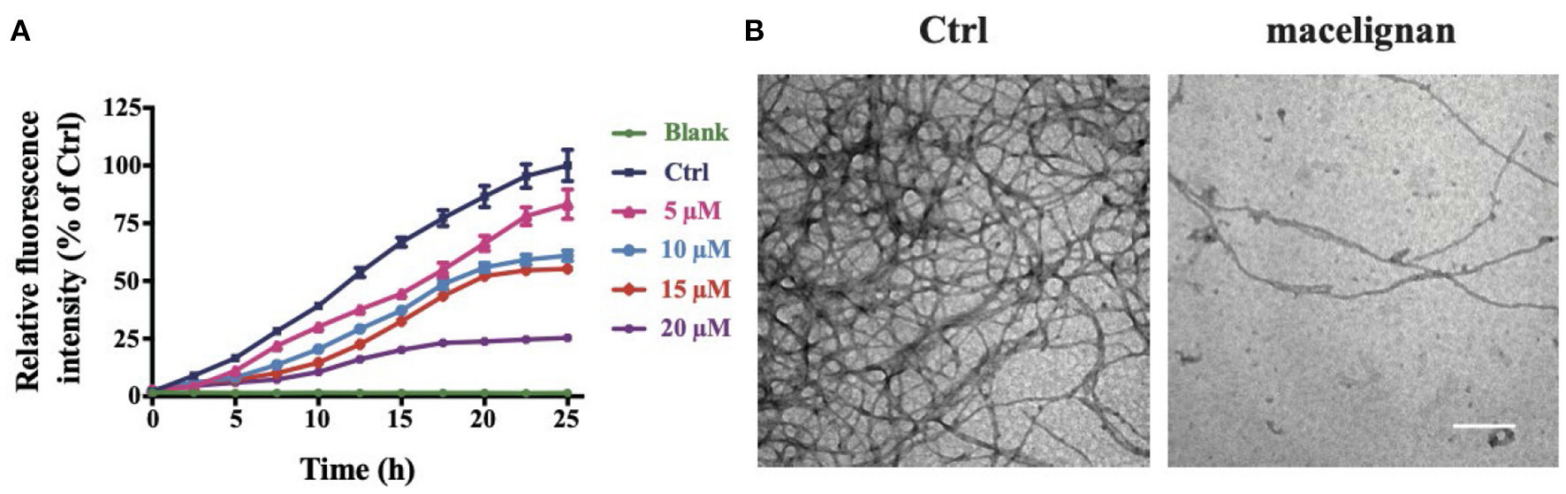
Macelignan (MLN) inhibits Tau aggregation *in vitro*. **(A)** The effects of MLN on real-time ThT fluorescence intensity detection of R3-Tau. **(B)** The effects of MLN (20 μM) on R3-Tau aggregation by TEM images. Scale bar = 500 nm.

Tau can be induced to form Tau fibers by heparin sodium *in vitro*, thus imitating the nerve fiber tangles formed by abnormal aggregation of Tau *in vivo*. Thioflavin T could bind to the beta structure of nerve fibers to emit fluorescence. In order to investigate the effects of MLN on Tau aggregation intuitively, we used the fluorescence assay of ThT and TEM. As shown in Figure 1A, the intensity of fluorescence of R3-Tau increased as time progress. In contrast, the intensity of fluorescence in the MLN treatment group was significantly suppressed in a concentration-dependent manner. The inhibition of R3-Tau aggregation by MLN could be more directly observed with TEM analysis. The TEM images confirmed that the nerve fiber tangles in the MLN were much less than that in the control (Figure 1B). Results of TEM and ThT experiments indicated that MLN had a suppression effect on Tau aggregation.

### MLN Reduced Tau Phosphorylation in Tau Overexpressing Cells and 3 × Tg-AD Mouse Primary Neuron Cells

**Figure 2:**
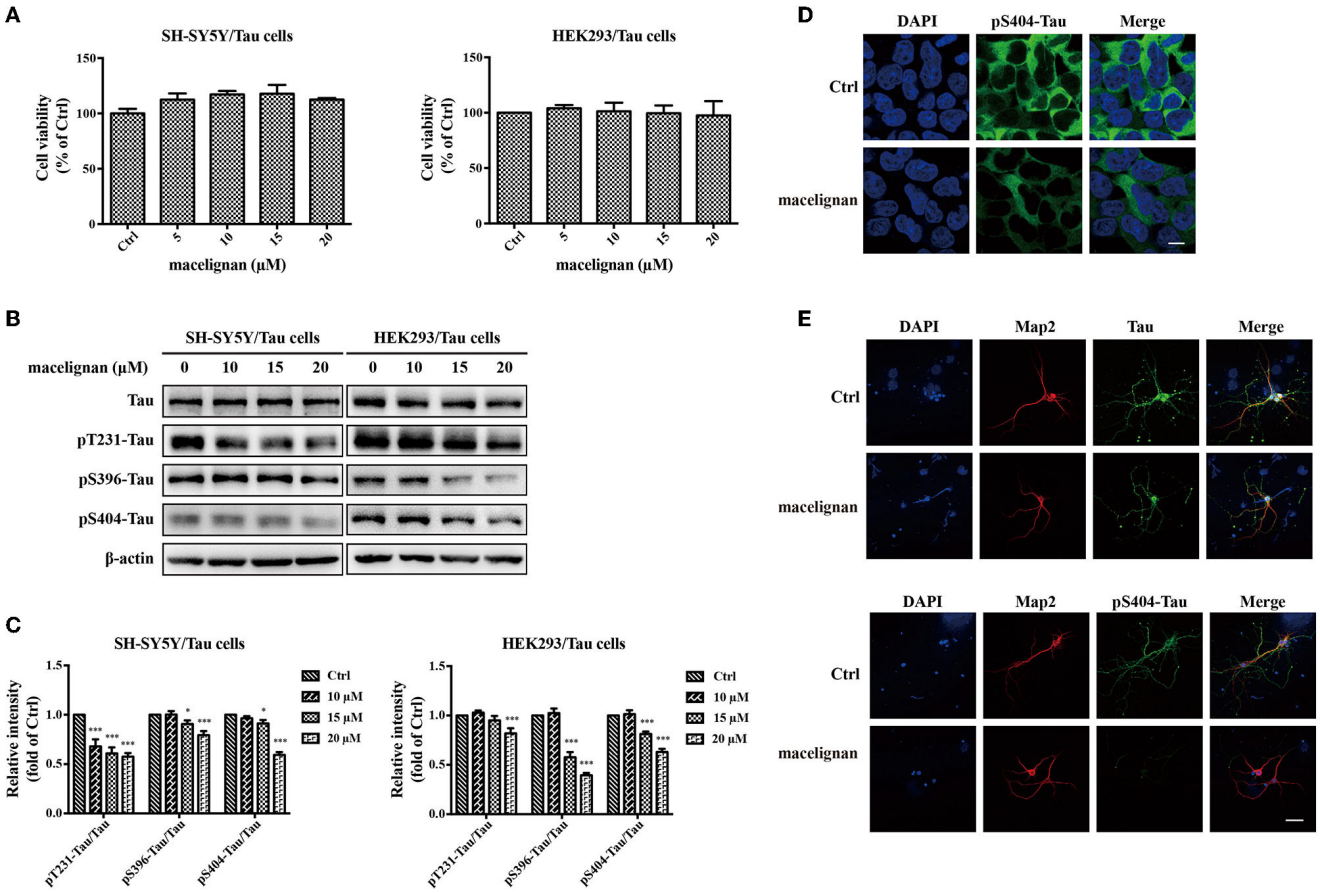
Macelignan (MLN) inhibits Tau phosphorylation in Tau overexpressing cells and 3× Transgene-AD mouse primary neuron cells. **(A)** SH-SY5Y/Tau and HEK 293/Tau cells were incubated in serial concentrations of MLN (0, 5, 10, 15, and 20 μM) for 24 h and then the effect of MLN on cell viability was measured by the CCK-8 assay. **(B)** The effect of MLN on the protein levels of Tau, pS396-Tau, pS404-Tau and pT231-Tau in Tau over-expressing cells were determined by Western blot analysis. **(C)** The protein relative intensity of Tau, pS396-Tau, pS404-Tau, pT231-Tau in Tau over-expressing cells was shown. β-actin was used as a loading control in the Western blot analysis. All results were from independent triplicate experiments, *p < 0.05, **p < 0.01, and ***p < 0.001 vs. control. Immunofluorescence staining was used to show the effect of MLN on Tau and its phosphorylation. DAPI (in blue) was used to stain the nuclei. Map2 (in red) and Tau or pS404-Tau (in green) were stained using their antibodies. **(D)** The staining results in HEK/293 cells (scale bar=10 μm) were shown. **(E)** The staining results in primary neuron cells (Scale bar=50 μm) were shown.

SH-SY5Y/Tau and HEK293/Tau cells are two nerve cell lines that could overexpress Tau. 3× Tg-AD mouse primary neurons are able to fully simulate the pathological features of AD, hence are considered to be the ideal cell model for studying AD. In this study, the effects and the mechanism of MLN on Tau phosphorylation were investigated by using these cells. After being incubated with different concentrations (0, 5, 10, 15, 20 μM) of MLN for 24 h, the cell viability assay showed that MLN were not cytotoxic to both SH-SY5Y/Tau and HEK293/Tau cells (Figure 2A). Total Tau and phosphorylated Tau in SH-SY5Y/Tau, HEK293/Tau, and 3× Transgene-AD mouse primary neuron cells were measured. As shown in Figures 2B,C, the protein levels of pS396-Tau/Tau, pS404-Tau/Tau, and pT231-Tau/Tau were substantially diminished at a dose-dependent manner in Tau over-expressing cells. The immunofluorescence staining results were consistent with that of western blot (Figure 2D), which confirmed that MLN had an inhibitory effect on pS404-Tau expression in HEK293/Tau cells. Results in the 3× Transgene-AD mouse primary neurons were similar to that of Tau over-expressing cells. After treatment with 20 μM MLN, the intensity of green fluorescence, which represented the levels of pS404-Tau, in primary neuron cells was decreased significantly, and the total Tau showed no significant change, which suggests that MLN treatment can reduce phosphorylated Tau levels (Figure 2E).

### MLN Promoted PP2A Activity in Tau Overexpressing Cells

**Figure 3:**
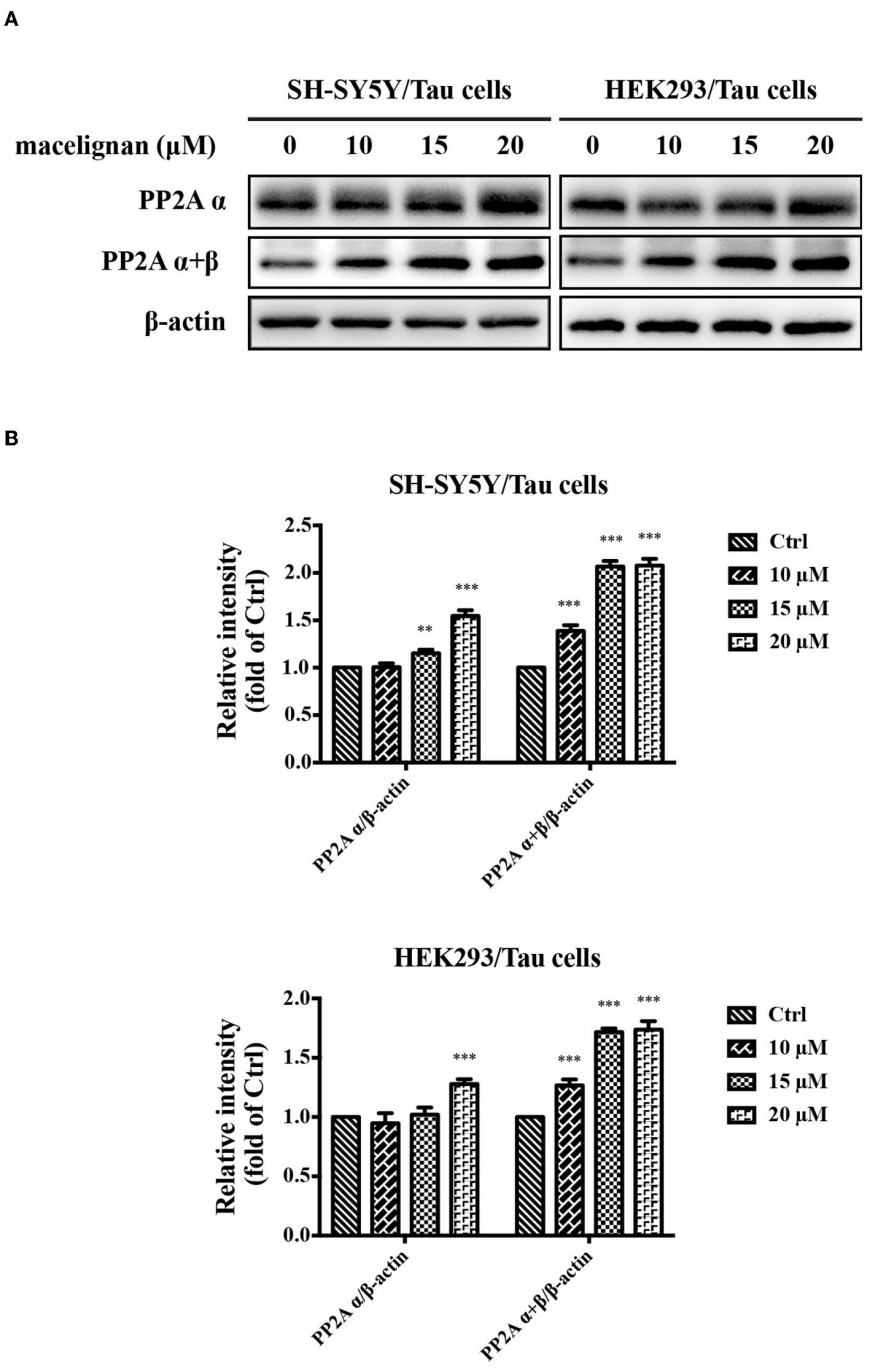
Macelignan (MLN) promotes PP2A activity in Tau overexpressing cells. **(A)** SH-SY5Y/Tau and HEK293/Tau cells were added with series concentrations of MLN (0, 10, 15 and 20 μM) for 24 h, and then the effects of MLN on the levels of PP2A α and PP2A α + β were determined using Western blot analysis. **(B)** The protein relative intensity of PP2A α and PP2A α + β in Tau over-expressing cells were shown. β-actin was used as a loading control in the Western blot analysis. All results were from three independent experiments, *p < 0.05, **p < 0.01, and ***p < 0.001 vs. control.

PP2A is a kind of phosphatase that regulates the phosphorylation of Tau, which is closely related to the Alzheimer's disease (22). Studies have shown that sodium selenate could enhance PP2A activity and improve cognitive impairment in a PP2A-dependent manner in AD models with the aid of dysregulating Tau phosphorylation (23). The effects of MLN on PP2A activity was investigated in this study. Results were shown in Figures 3A,B. PP2A α and PP2A α+β activities were significantly enhanced at a concentration-dependent manner. In general, MLN treatment promotes PP2A activity in Tau over-expressing cells.

### MLN Enhanced Autophagy in Tau Overexpressing Cells

**Figure 4:**
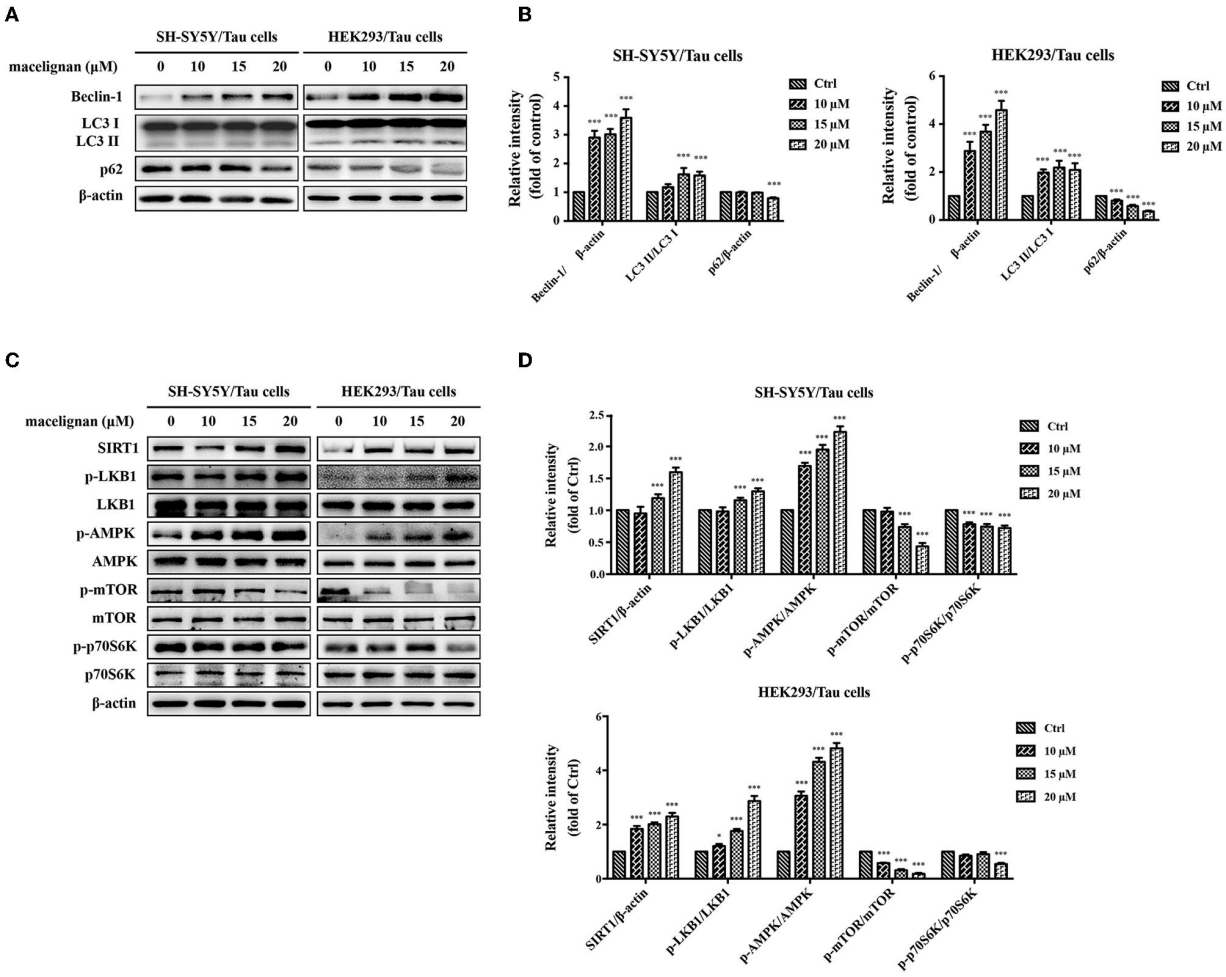
Macelignan (MLN) enhances autophagy in Tau overexpressing cells. SH-SY5Y/Tau and HEK293/Tau cells were incubated with series concentrations of MLN (0, 10, 15 and 20 μM) for 24 h and then their effects on the levels of autophagy-related proteins (Beclin-1, LC3, p62) were measured by Western blot analysis and presented in **(A)**. The differences of relative intensity of those proteins were shown in **(B)**. Effects of MLN on the AMPK/mTOR pathway-related protein (SIRT1, LKB1, AMPK, mTOR, p70S6K) were measured by Western blot and shown in **(C)**. The differences of relative intensity of those proteins were shown in **(D)**. β-actin was used as a loading control in the Western blot analysis. All results were from three independent experiments, *p < 0.05, **p < 0.01, and ***p < 0.001 *vs*. control.

Autophagy is an essential degradation pathway in mammalian cells to eliminate abnormal protein aggregation and accountable for protein homeostasis and neuronal health. Autophagy is associated with many neurodegenerative diseases (24). Beclin-1, LC3 and p62 are usually regarded as specific markers of autophagy, and the adjustments in their expression are often associated with the changes in autophagy. In this study, the effects of MLN on autophagy was investigated. As shown in Figures 4A,B, the expressions of Beclin-1 and LC3 II/LC3 I increased significantly by MLN treatment, while the expression of p62 decreased. These autophagy-related protein expression adjustments demonstrated that MLN could activate autophagy.

### MLN Activated the AMPK/MTOR Signal Pathway to Enhance Autophagy

To understand the mechanism by which MLN activates autophagy, the related signal pathways were investigated. Cell extracts from SH-SY5Y/Tau and HEK293/Tau were analyzed using Western blot for the expression and phosphorylation level of AMPK, mTOR, and p70 S6K. As shown in Figure 4C, the protein levels of p-AMPK increased in a dose-dependent manner with MLN treatment. The phosphorylation of mTOR and p70 S6K were dose-dependently reduced. However, the expressions of AMPK, mTOR, and p70 S6K showed no significant changes. These changes indicated that MLN could upregulate the AMPK/mTOR signal pathway to promote autophagy. AMPK is a vital nutrient sensor. The changes in AMPK phosphorylation are always linked to energy metabolism. Thus, the variations in the AMPK upstream regulation proteins SIRT1 and LKB1 were examined. As shown in Figures 4C,D, the protein levels of SIRT1 and p-LKB1 increased when treated with MLN. In summary, after MLN treatment, the AMPK/mTOR signal pathway in SH-SY5Y and HEK293/Tau cells were upregulated, which activated the cellular level of autophagy.

### MLN Enhanced PERK/eIF2α Signal Pathway to Decline Aβ Aggregation in N2a/SweAPP Cells

**Figure 5:**
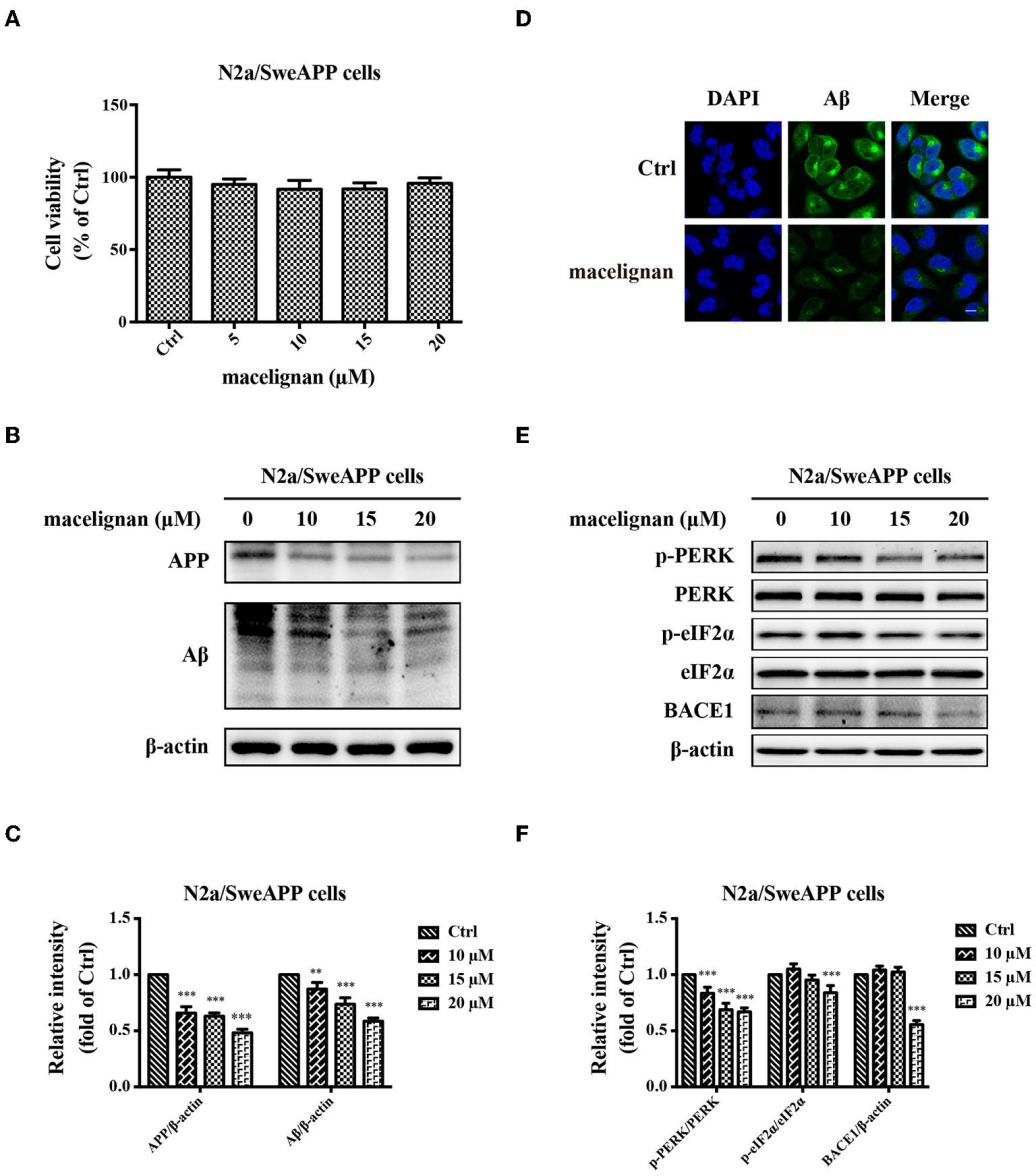
Maceglinan (MLN) decreases Aβ production in N2a/SweAPP cells. **(A)** The effects of macelignan on N2a/SweAPP cell viability were measured by CCK-8 assay. **(B)** N2a/SweAPP cells were added with series concentrations of MLN (0, 5, 10, 15 and 20 μM) for 24 h and then the effects of MLN on the expression of APP and Aβ were determined by Western blot analysis, and the differences in protein relative intensity were shown in **(C)**. **(D)** Immunofluorescence staining was used to show the effects of MLN on Aβ. DAPI (in blue) was used to stain the nuclei, and Aβ (in green) was stained by using Aβ antibody. Scale bar=10 μm. **(E)** eIF2α and PERK were analyzed by using Western blot assay. And **(F)** The differences of relative intensity of p-PERK/PERK, p-eIF2α/eIF2α, and BACE1 bands were shown. β-actin was used as a loading control in the Western blot analysis. All results were from independent triplicate experiments, *p < 0.05, **p < 0.01, and ***p < 0.001 vs. control.

Since Aβ deposition is one of pathogenesis in AD, the effects of MLN on Aβ in N2a/SweAPP cells were investigated. After incubating with one of the concentrations (0, 5, 10, 15, 20 μM) of MLN for 24 h, N2a/SweAPP cell viability was measured, which indicated that MLN was not cytotoxic (Figure 5A). Then, the expressions of Aβ and APP in N2a/SweAPP were examined using Western blot assay. Results showed that MLN decreased APP and Aβ expression in a dose-dependent manner (Figures 5B,C). Immunofluorescence staining results were consistent with that of Western blot, which confirmed that MLN had a downregulation effect on Aβ (Figure 5D). Next, the mechanism of Aβ inhibition was explored. Considering that BACE1 is the key rate-limiting enzyme (25) accountable for Aβ deposition and the PERK/eIF2α signal pathway is highly drawn into BACE1 translation (26), whether MLN regulates PERK/eIF2α signal pathway to reduce Aβ deposition was investigated. As shown in Figures 5E,F, the phosphorylation of PERK and eIF2α in N2a/SweAPP cells decreased after MLN treatment. Taken together, the Aβ deposition, expression of APP, and phosphorylation of PERK and eIF2α were all decreased in a dose-dependent manner in N2a/SweAPP cells with the treatment of MLN.

## Discussion

Recent statistics show that 6.2 million Americans have AD and will increase to 13.8 million by 2060 (27). Although some clinical drugs can alleviate the symptoms of AD, there is no cure. In the present research, it is found that MLN had a good anti-AD potential through reducing Tau phosphorylation and decreasing Aβ aggregation.

Here, it was observed that MLN could decrease the R3-Tau's ThT fluorescence intensity at a dose-dependent manner and inhibit the aggregation of Tau *in vitro*, which were comparable to the results of a previous study (28), suggesting MLN has neuroprotective effects. It was additionally noticed that the phosphorylation of Tau was significantly reduced at Ser 404 site in primary cortical neurons and a significant decrease in phosphorylated Tau at Ser 396, Ser 404, and Thr 231 site in SH-SY5Y/Tau and HEK293/Tau cells in response to MLN treatment. Nerve fiber tangles caused by abnormally excessive ranges of phosphorylation of Tau are one of the typical pathological aspects of AD (2). It is known that under normal circumstances, Tau exists in neurons, induces the microtubule bunching, maintains microtubule stability, and normalize axonal transport of nerves (29). When hyperphosphorylation happens to Tau, its bound affinity to microtubules and depolymerization decrease (30). It has been reported that decreasing Tau hyperphosphorylation has been considered as an approach to improve cognition (31). Our present study found that MLN could reduce the expression of Tau phosphorylation, which may further inhibit Tau aggregation and the formation of nerve fiber tangles.

It has been suggested that a number of post-translational changes of Tau play an important function in the aggregation of Tau linked to AD, and Tau phosphorylation is the primary one amongst those post-translational modifications (32). The disruption of the balance between Tau kinase and phosphatase activities is considered the cause of the unusual Tau phosphorylation (33). Decrease in phosphatase activity is related to ordinary phosphorylation and aggregation of Tau in AD (34). PP2A is a kinase related to Tau phosphorylation, consisting of a structural A subunit (α and β subtypes), a highly variable regulatory subunit B, and a catalytic C subunit (α and β subtypes) (35, 36). Previous studies have proven that the inhibition of PP2A activity results in Tau hyperphosphorylation and spatial memory deficiency (37), which indicates that PP2A might be a therapeutic target for AD. In the present study, we demonstrated that the treatment of MLN could enhance the activity of PP2A, which may contribute to the downregulation of Tau phosphorylation.

Previous studies have proven autophagy can manage the renewal of soluble cytoplasmic proteins, as a result decreasing the accumulation of atypical proteins, which prevents neurodegenerative diseases (11, 38, 39). Further studies have found that the expression of Beclin-1 is significantly reduced in Beclin-1-deficient transgenic mice, which leads to a decrease of autophagy in neurons which is the reason for neurodegenerative diseases (40). LC3 is one of the biomarkers of autophagy, which is crucial for forming autophagosomes during autophagy (41). Another biomarker of autophagy, p62, can interact with LC3 to remove protein aggregation (42). Our research found that levels of LC3 and Beclin-1 were increased, and p62 was decreased in SH-SY5Y/Tau and HEK293/Tau cells after the treatment of MLN. AMPK is a vital energy sensor in cells. Some studies found that the downregulation of AMPK may relate to neurodegenerative diseases such as AD (43). Our results showed that MLN could upregulate AMPK phosphorylation, further downregulating the expression of p-mTOR and p-p70S6K to activate autophagy in Tau overexpressing cells. Previous studies found that the activation of SIRT1 in the brain could modulate amyloid neuropathology in the AD brain (44), and other studies also indicated that SIRT1 could regulate the AMPK/mTOR pathway (45). LKB1 is an upstream enzyme of AMPK, which regulates the activation of AMPK through phosphorylation (46). Our results showed that MLN could stimulate SIRT1 to activate LKB1 phosphorylation, which further promotes phosphorylated AMPK expression. This suggests that MLN could stimulate SIRT1 and LKB1, the upstream enzymes of AMPK. In turn, this results in AMPK activation to improve cellular energy metabolism, which promotes autophagy to decrease the hyperphosphorylation of Tau. All those ultimately prevent phosphorylated Tau to form NFTs.

Recently, multi-target strategy against AD has become a research focus. We thereby hypothesize that apart from Tau phosphorylation inhibition, MLN may have an effect on amyloid plaques as well. To explore this effect, we used N2a/SweAPP cells, which overexpress APP as well as BACE1 and Aβ. We showed that MLN could decrease Aβ and APP expression in N2a/SweAPP cells. Endoplasmic reticulum performs many critical cellular functions in the body, including protein homeostasis and lipid formation. When proteins in the endoplasmic reticulum are misfolded, they trigger endoplasmic reticulum stress, which in response makes phosphorylated eIF2α to induce the timely closure of protein synthesis to protect cells (47). However, hyperphosphorylation of eIF2α can persistently inhibit the translation of protein synthesis, which may lead to synaptic failure, accompanied by abnormal expression of synaptic proteins, and ultimately cause neurodegenerative changes and memory deficits (48). A previous study verified that *Thamnolia vermicularis* extracts could diminish the phosphorylation of PERK and eIF2α in CHO-APP/BACE1 cells and astrocytes, which indicates that the response to the misfolded protein is involved in the amyloid plaque formation (49). Our results are consistent with the previous findings, showing that MLN can decrease the phosphorylation of PERK and eIF2α to reduce BACE1 expression. Therefore, we conclude that MLN can activate the misfolded protein response-related signal pathway to reduce BACE1 translation, which further inhibits the cleavage of APP and ultimately suppresses Aβ deposition.

**Figure 6:**
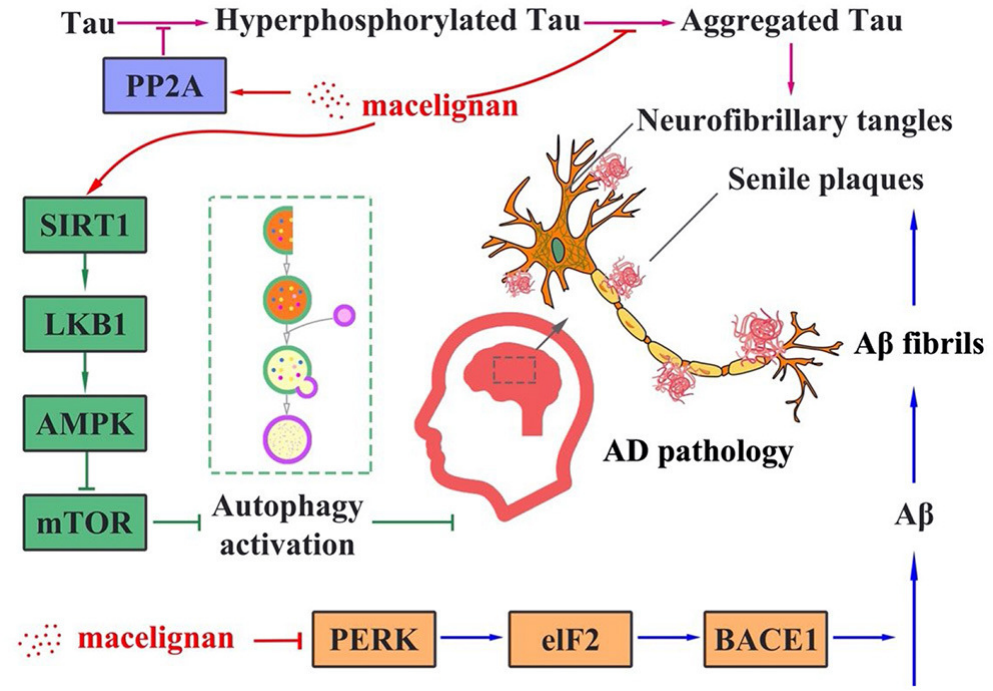
The proposed molecular mechanism of macelignan on anti-Alzheimer's Disease activity.

A previous study has demonstrated that MLN has a neuroprotective property by assuaging neuroinflammation (50). Different from this result, our results illustrate that MLN could improve AD via reduction of both Tau hyperphosphorylation and Aβ aggregation. Taken all results together, the proposed molecular mechanism for MLN in AD treatment is that MLN could activate PP2A and SIRT1/AMPK/mTOR signaling-mediated autophagy to reduce Tau phosphorylation. In the meantime, it also activates the PERK/eIF2α signal pathways to suppress Aβ deposition. The summarized molecular mechanism is shown in Figure 6.

## Conclusion

In summary, our results show that MLN can inhibit both Tau hyperphosphorylation and Aβ production, which indicates that MLN has the potential to be developed as a treatment for AD.

## Data Availability Statement

The original contributions presented in the study are included in the article/supplementary material, further inquiries can be directed to the corresponding authors.

## Author Contributions

XX and JL: conceptualization and supervision. LG, NC, ML, and DB: data curation. LG, NC, LY, WF, and YW: formal analysis. ZH, XX, and JL: funding acquisition. LG, NC, QL, and ZL: investigation. LG, NC, and XX: methodology and writing–original draft. ZH, QL, XX, and JL: resources. JL: writing–review and editing. All authors gave final approval and agreed to be accountable for all aspects of the work.

## Funding

This work was supported by the National Natural Science Foundation of China (32172193, 31970366, and 41876188), National Key R&D Program of China (2018YFD0901106 and 2018YFA0902500), the Science and Technology Innovation Commission of Shenzhen (JCYJ20190808141415052), Medical Scientific Research Foundation of Guangdong Province of China (A2021219), and Grant Plan for Demonstration City Project for Marine Economic Development in Shenzhen (No. 86). Royal Society of New Zealand Catalyst Seeding Fund (21-AUT-005-CSG).

## Conflict of Interest

The authors declare that the research was conducted in the absence of any commercial or financial relationships that could be construed as a potential conflict of interest.

## Publisher's Note

All claims expressed in this article are solely those of the authors and do not necessarily represent those of their affiliated organizations, or those of the publisher, the editors and the reviewers. Any product that may be evaluated in this article, or claim that may be made by its manufacturer, is not guaranteed or endorsed by the publisher.

## Abbreviations

AD: Alzheimer's disease
Aβ: amyloid β-protein
APP: β-amyloid precursor protein
AMPK: AMP-activated protein kinase
BSA: bovine serum albumin
CCK: cell counting kit
DAPI, 4′, 6-Diamidino-2-Phenylindole: Dihydrochloride
DMEM: Dulbeco's modified Eagle's medium
FBS: fetal bovines serum
FDA: United States Food and Drug Administration
HEK293: human embryonic kidney 293
HRP: orseradish peroxidase
LC3: microtubule-associated protein II light chain3
LKB1: liver kinase B1
MLN: macelignan
mTOR1: mammalian target of rapamycin
NC: nitrocellulose filter
NFTs: neurofibrillary tangles
PBS: phosphate-buffered saline
PFA: paraformaldehyde
p-AMPK: phospho-AMPK
p-LKB1: phospho-LKB1
RIPA: radioimmunoprecipitation assay
SIRT1: silent information regulator of transcription 1
SPF: specific pathogen-free
ThT: thioflavin T.
